# Ingested Fish Bone: An Unusual Mechanism of Duodenal Perforation and Pancreatic Trauma

**DOI:** 10.1155/2012/308510

**Published:** 2012-08-05

**Authors:** Dimitrios Symeonidis, Georgios Koukoulis, Ioannis Baloyiannis, Apostolos Rizos, Ioannis Mamaloudis, Konstantinos Tepetes

**Affiliations:** Department of General Surgery and Radiology, University Hospital of Larissa, Mezourlo, 41110 Larissa, Greece

## Abstract

Ingestion of gastrointestinal foreign bodies represents a challenging clinical scenario. Increased morbidity is the price for the delayed diagnosis of complications and timely treatment. We present a case of 57-year-old female patient which was admitted in the emergency room department complaining of a mid-epigastric pain over the last twenty-four hours. Based on the patient's history, physical examination and elevated serum amylase levels, a false diagnosis of pancreatitis, was initially adopted. However, a CT scan confirmed the presence of a radiopaque foreign body in the pancreatic head and the presence of air bubbles outside the intestinal lumen. The patient was unaware of the ingestion of the foreign body. At laparotomy, after an oblique duodenotomy, a fish bone pinned in the pancreatic head after the penetration of the medial aspect of the second portion of the duodenal wall was identified and successfully removed. The patient had an uneventful postoperative recovery. Wide variation in clinical presentation characterizes the complicated fish bone ingestions. The strategically located site of penetration in the visceral wall is responsible for the often extraordinary gastrointestinal tract injury patterns. Increased level of suspicion is of paramount importance for the timely diagnosis and treatment.

## 1. Introduction

Foreign body ingestion is a common phenomenon especially in the pediatric population. The vast majority of these pediatric ingestions are accidental. *Intentional ingestion of foreign bodies especially occurs in individuals beyond the age of adolescence* [1, 2]. While coins, toy's parts and batteries are the most commonly encountered objects in children, meat and fish bones represent the most often accidentally ingested foreign bodies in adults [2]. The size, shape, and the material of the foreign body as well as the patient's age determine the natural history of this condition. Sharp foreign bodies increase the risk for complications and the possibility of successful observational management declines. The incidence of foreign bodies requiring operative removal varies greatly in the literature. Figures ranging from 1% to 14% have been reported [3].

A conservative approach to foreign body ingestions is generally justified, although early endoscopic removal of objects within the stomach is recommended. The success of nonoperative management depends on the absence of symptoms in a patient with a clear history [4]. On the other hand, sharp object ingestions warrant a higher index of suspicion as they are associated with a significantly higher risk of perforation [3]. Terminal ileum, sigmoid colon, and rectum are the most frequent perforation sites [5]. Patients are usually unaware of the foreign body ingestion rendering preoperative diagnosis a real challenging process. The time interval between the ingestion and the possible perforation can be rather prolonged, that is, more than 10 days making the “cause and effect” correlation between the two events especially difficult [5].

With the notable exception of the *esophagus*, the upper gastrointestinal tract is not generally considered a usual site of perforation due to ingested sharp foreign bodies. In the present study, we present an interesting case of duodenal perforation caused by an ingested fish bone. Interestingly, the foreign body that penetrated the duodenal wall was dispersed and pinned in the anatomically adjacent pancreatic head representing a real infrequent and extraordinary mechanism of penetrating pancreatic trauma.

## 2. Case Presentation

A 57-year-old, otherwise healthy, female patient was admitted in the emergency room department complaining of midepigastric pain over the past twenty-four hours. The onset of pain was relatively gradual, two hours after a heavy meal. Nausea was present from the outset and two episodes of bile stained vomiting offered a temporal relief from the pain. The consistency of the pain as well as *an increased body temperature* (up to 37,8 degrees celsius) compelled the patient to seek medical assistance.

On arrival, the patient had a body temperature of 37,5 degrees celsius, blood pressure of 120/75 mm Hg, pulse rate of 105/min, and respiratory rate of 17 breaths/min. On physical examination, the abdomen was relatively soft with however, notable tenderness elicited during the deep palpation of the midepigastrium. Signs of parietal peritoneum irritation such as rebound tenderness as well as pain during abdominal percussion were not observed. The patient had a blood gas ph. of 7.35, C-reactive protein levels of 11 mg/l while white blood cell (WBC) count were 14000/mm^3^. Serum amylase levels were elevated up to 500 U/dL, while *liver enzyme serum levels* were within normal range.

**Figure 1:**
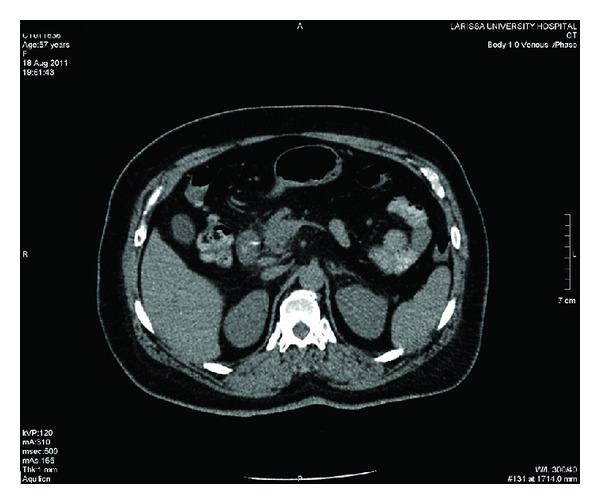
Contrast-enhanced CT image showing the foreign body in the duodenum as well as the presence of air outside the lumen.

**Figure 2:**
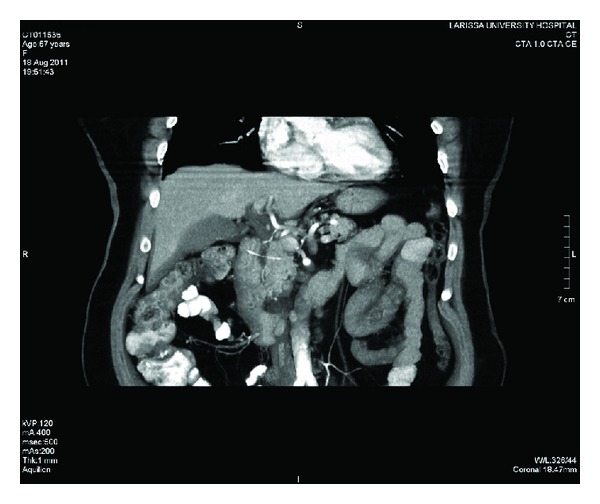
Contrast-enhanced CT image showing the radiopaque foreign body penetrating into the pancreatic head.

The patient was submitted to an abdominal ultrasound that ruled out cholelithiasis as well as any gross pathology emanating from the extrahepatic bile ducts. With a suspected diagnosis of pancreatitis, despite the absence of obvious predisposing factors, the patient was admitted to the department's clinic for observational and supportive management. However, as the patient's condition did not ameliorate after two days of conservative treatment a computed tomography scan (CT) of the abdomen was decided. Surprisingly, a radiopaque foreign body in the pancreatic head, *which the patient was not aware of ingesting*, in continuity with the duodenum as well as the presence of air bubbles in the area of question suggestive of a probable intestinal perforation was revealed. See Figures 1 and 2.

An emergency operation was decided and a laparotomy via a midline vertical supraumbilical incision was undertaken. At laparotomy, peritoneal cavity appeared clear without evidences of gross contamination. After the mobilization of the right colonic flexure, the duodenum was clearly visualized. Then, *a Kocher manoeuvre was performed to ensure the adequacy of duodenal inspection*. An induration at the second portion of the duodenum and the adjacent area of the pancreatic head was observed during palpation without, however, any identifiable perforation. An oblique—in relation to the luminal axis of the duodenum—duodenotomy was performed and a sharp thin foreign body consistent with fish bone was subsequently identified. The bone was pinned at the medial-posterior wall of the 2nd portion of the duodenum just adjacent to the ampulla of Vater with an orientation towards pancreatic head. After the successful removal of the fish bone with gentle traction the duodenotomy was then closed in two layers with interrupted 3-0 absorbable suture material. 

The patient had an uneventful postoperative period and was discharged from the hospital on the 7th postoperative day. A scheduled followup at the outpatient clinic of our department thirty (30) days after the procedure confirmed the absence of any postoperative complication.

## 3. Discussion

Gastrointestinal foreign bodies *represent a challenging clinical scenario* in the setting of the emergency department. Increased morbidity and mortality are the price for the delayed diagnosis of complications and subsequent timely treatment [6]. Foreign bodies' perforations of the stomach and duodenum tend to present with a longer and more innocuous clinical picture than perforations located in the jejunum or ileum. In addition, the former locations are usually associated with the development of an abdominal mass or abscess and tend to cause less systematic signs of infection [7]. Increased level of suspicion and immediate correlation between the history of ingestion and the physical findings are the cornerstones for prompt diagnosis. Imaging studies are by definition invaluable in this direction. Provided that a precise history of foreign body ingestion is not always attained, the use of modern CT is of paramount importance; especially in cases where a complication such as perforation has supervene [8].

However, when fish bone becomes the foreign body of interest the field becomes more obscure. Prediction of the presence of fish bones by symptoms or radiograph is quite poor and usually misleading [9]. In such instance, the contribution and accuracy of CT in the preoperative diagnosis of a fish-bone-associated perforation is questioned and a correct preoperative diagnosis is seldom possible [10]. Regarding treatment either watchful waiting or endoscopic removal in the specific cases of lodged fish bones in the pharynx or the oesophagus seems as the reasonable approach [9]. However, when perforation complicates the clinical picture emergency operative intervention is warrant.

In the present study, we present the rare case of a middle-aged female patient suffering from a duodenal perforation from a fish bone penetrating the duodenal wall and the head of the pancreas. Based initially on clinical history, physical examination and laboratory test results, an initial false diagnosis of pancreatitis, were adopted. The increased serum levels of amylase in the preoperative setting proved especially misleading confirming the enzyme's limitations regarding the diagnosis of acute pancreatitis. However, it was the CT scan findings that ruled out the diagnosis of pancreatitis and oriented the diagnostic thinking toward the correct direction. The recognition of the offending radiopaque foreign object as well as the indirect signs of perforation, that is, air outside the gastrointestinal tract was diagnostic. The course of the fish bone and the penetration of the pancreatic head through the lumen of the duodenum as depicted either by the preoperative imaging as well as by laparotomy findings highlighted this real extraordinary mechanism of pancreatic trauma. Nevertheless, the patient admitted unawareness of the ingestion of the foreign body; however, a fish meal four days before the admission was elicited from patient's history.

With a course origin in the medial aspect of the duodenal wall just adjacent to the ampulla of Vater, this sharp object penetrated into the pancreatic parenchyma. The perforation did not communicate freely with the entire peritoneal cavity explaining the subtle clinical presentation. The clinical hallmarks of a perforated hollow viscous, that is, rigidity, rebound tenderness were absent. This walled-off process with a foreign body present, however, elicited either a localized inflammatory reaction powered with the microbial burden of the upper gastrointestinal tract as well as a systematic response manifested with fever, leucocytosis and C-reactive protein level elevation. 

 Regarding the surgical technique, we used an obliquely oriented in relation to the luminal axis of the duodeno-duodenostomy in order to achieve optimal exposure. In addition, the closure of this type of duodenotomy represents a combination between the Heinecke-Miculicz technique and the simple longitudinal closure limiting the incidence of subsequent stenosis. The satisfactory closure result rendered the addition of a gastrojejunostomy unnecessary. No additional manoeuvre besides the simple gentle removal of the fish bone was undertaken for the site of perforation.

In conclusion, wide variation in clinical presentation characterizes the complicated fish bone ingestions. The strategically located site of penetration in the visceral wall is responsible for the often extraordinary gastrointestinal tract injury patterns. Increased level of suspicion is of paramount importance for the timely diagnosis and treatment.
